# Plants-Derived Neuroprotective Agents: Cutting the Cycle of Cell Death through Multiple Mechanisms

**DOI:** 10.1155/2017/3574012

**Published:** 2017-08-22

**Authors:** Taiwo Olayemi Elufioye, Tomayo Ireti Berida, Solomon Habtemariam

**Affiliations:** ^1^Department of Pharmacognosy, Faculty of Pharmacy, University of Ibadan, Ibadan, Nigeria; ^2^Pharmacognosy Research Laboratories and Herbal Analysis Services, University of Greenwich, Chatham-Maritime, Kent ME4 4TB, UK

## Abstract

Neuroprotection is the preservation of the structure and function of neurons from insults arising from cellular injuries induced by a variety of agents or neurodegenerative diseases (NDs). The various NDs including Alzheimer's, Parkinson's, and Huntington's diseases as well as amyotropic lateral sclerosis affect millions of people around the world with the main risk factor being advancing age. Each of these diseases affects specific neurons and/or regions in the brain and involves characteristic pathological and molecular features. Hence, several in vitro and in vivo study models specific to each disease have been employed to study NDs with the aim of understanding their underlying mechanisms and identifying new therapeutic strategies. Of the most prevalent drug development efforts employed in the past few decades, mechanisms implicated in the accumulation of protein-based deposits, oxidative stress, neuroinflammation, and certain neurotransmitter deficits such as acetylcholine and dopamine have been scrutinized in great detail. In this review, we presented classical examples of plant-derived neuroprotective agents by highlighting their structural class and specific mechanisms of action. Many of these natural products that have shown therapeutic efficacies appear to be working through the above-mentioned key multiple mechanisms of action.

## 1. Introduction

Neuroprotection is a term used to refer to strategies and relative mechanisms that shield the central nervous system (CNS) from neuronal injuries caused by chronic (e.g., Alzheimer's and Parkinson's diseases) or acute (e.g., stroke) neurodegenerative diseases (NDs) [1]. These acute or chronic diseases result from breakdown and deterioration of neurons of the CNS and often result in the deterioration of the cognitive as well as the intellectual faculties of the sufferers. The onset of NDs symptoms is usually gradual as well as progressive and includes loss of memory, primarily short-term, difficulty in learning, motor coordination, and many other functional loses [2, 3]. Ageing, defined as a complex physiological process involving both morphological and biochemical changes that progressively unfold as we get older [1], has been found to be closely associated with NDs. Ageing stands out as a major risk factor among the other etiological factors of NDs, including hypertension, genetic and/or environmental factors, and infections. With increasing age, aggregation of proteins, inflammation, oxidative stress, and loss of neurotransmitters, which are common to the pathology of NDs, also occur more often [1, 4, 5].

Nature remains to be a veritable source of medicines to mankind. Many important drugs such as vincristine, artemisinin, and gentamicin, which are still in use today, are obtained from natural sources or are designed on structural fingerprints of naturally occurring molecules [6, 7]. Furthermore, traditional medicine remains a vital alternative source of medicine all over the world today with some estimate suggesting to account to about 80% of the primary health care system in some developing countries (e.g., Nigeria, Ghana, China, and India [8, 9]). The increasing incidence of resistance (especially to antibiotics and antimalarials), undesirable side effects, high cost, and lack of efficacy after prolonged usage of the existing drugs in use has led to a renewed interest in the development of new drug candidates from natural sources [10, 11]. For example, drugs such as amantadine, memantine, donepezil, selegiline, galantamine, and rivastigmine that are clinically available for the management of NDs are only able to provide symptomatic relief and slow the progression of the diseases [12, 13]. These drugs, such as the synthetic donepezil, are also associated with side effects [14]. Hence, a great deal of research focus has been given in recent years to herbs and other natural products used in ethnomedicine around the world for age-related CNS diseases. 

Numerous natural products, but primarily plants extracts, have been reported to be used in traditional medicine for neuroprotective, memory enhancing, and antiageing purposes. Examples of such plants include* Ginkgo biloba, Panax ginseng, Curcuma longa, Bacopa monnieri, *and* Salvia officinalis* [1, 15]. These plants have been studied to confirm the traditional claim with special attention given in understanding the mechanism by which they elicit the neuroprotective effects. This review is designed to give a brief description of a number of neurodegenerative diseases, the most important pathological events associated with the diseases, and an overview of therapeutic options with some popular plant-derived neuroprotective agents.

## 2. Overview of Neurodegenerative Diseases

Neurodegenerative disease is a term used to refer to various conditions which occur as a result of neuronal cell death, particularly, those of the CNS [16]. This deterioration is often associated with gradual onset of progressive symptoms, a major symptom being loss of memory. The NDs include Alzheimer's disease (AD), Parkinson's disease (PD), Lewy body dementia, multiple sclerosis, amyotrophic lateral sclerosis (ALS), and spongiform encephalopathy [13]. Of these NDs, AD is the most prevalent, accounting for over 60–70% of all forms of dementia [17]. Protein aggregation, inflammation, excitotoxicity, oxidative stress, and neurotoxicity have been implicated in the pathophysiology of NDs [3].

### 2.1. Alzheimer's Disease

The AD is the most prevalent and devastating disorder of the NDs. It is an incurable disease of cognition and behavioral impairment that affects social and occupational activities and is also a leading cause of institutionalization in the elderly [1, 18]. Clinically, AD is characterized by a progressive and irreversible memory deficits, cognitive deterioration, and personality changes, with a mean duration of about 8.5 years between onset of clinical symptoms and death [17]. Memory impairments are first to appear in the early stage of the disease, after which motor and sensory functions are affected as the disease progresses. The onset of AD is usually above 65 years of age, with risk from this age doubling every 5 years. Hence, it has been suggested that the risk for AD for persons living into their eighties rises to 20–40% depending on a variety of factors such as population dynamics and geography [19]. As the world population continues to age in parallel with economic development, the number of people with NDs and the associated dementia also continues to increase [20]. This increase has in turn prompted an enormous increment in research interest and efforts on the discovery of new therapeutic agents for primary, auxiliary, or tertiary prevention of these diseases [21].

The pathological hallmark of AD is the accumulation of protein aggregates to form two major lesions, namely, neurofibrillary tangles (NFTs) and senile plaques. Senile plaques are composed of fibrillar amyloid *β* (A*β*) peptides produced by cleavage of the A*β* precursor protein (APP), whereas NFTs consist of hyperphosphorylated microtubule-associated tau protein [22]. The mechanisms by which A*β* peptide aggregates act to cause AD are thought to include induction of oxidative damage [23] as well as inflammation [24] and neurotoxicity [19]. It is now known that the dysfunction of the central cholinergic system which plays a key role in the retrieval and storage of memory in the CNS is responsible for the cognitive deficit associated with AD [25–27]. Reports of substantial neocortical deficits in choline acetyltransferase (ChAT), the enzyme responsible for the synthesis of acetylcholine (ACh), discoveries of reduced choline uptake, ACh release, and loss of cholinergic perikarya from the nucleus basalis of Meynert, along with the emerging roles of ACh in learning and memory, led to the “cholinergic hypothesis of AD” [17].

### 2.2. Parkinson's Disease

The PD is the second most common ageing-related neurodegenerative diseases that can greatly impair quality of life with significant consequences in terms of cost of patient care [28]. Primarily a movement disorder, as opposed to AD which is mainly a cognitive disease, PD affects approximately 1% of the human population over the age of 60 [4]. Its classical signs include resting tremors, bradykinesia, extrapyramidal rigidity, and loss of postural reflexes such as disturbance in walking or equilibrium. The PD involves loss of dopaminergic neurons of the pars compacta region of the substantia nigra and their terminals in the corpus striatum [29]. Since neurodegeneration is not restricted to the basal ganglia, PD is also linked with nonmotor disorder like dementia. The association between PD and oxidative damage of neuronal cells has been well established. For example, the breakdown of dopamine (DA) by autooxidation has been shown to be linked to semiquinone metabolism and the generation of superoxide anion, hydrogen peroxide (H_2_O_2_), and monoamine oxidase (MAO) expression [19].

### 2.3. Other Neurodegenerative Diseases

Amyotrophic lateral sclerosis is thought to be caused by the mutation of the gene coding for the enzyme superoxide dismutase (SOD) and also by the misfolding of the same enzyme. The ALS is incurable and has generally a median survival of three years from onset to death. Its symptoms include tripping or stumbling when running, foot and wrist drop, slurred speech, and depression [30, 31]. Huntington disease (HD) is another incurable ND. It has an adult onset with autosomal dominant inherited disorder characterized by progressive brain degeneration, causing rapid deterioration and eventually death. Symptoms of the diseases include involuntary movement, dementia, and behavioral changes [32].

Prion diseases refer to a group of rare NDs caused by the aggregation of misfolded prion proteins. Prion proteins are known to be infectious and are presumed to cause some type of NDs referred to as spongiform encephalopathy: for example, Creutzfeldt-Jacob disease and* kuru* in humans,* Scrapie* in sheep, and bovine spongiform encephalopathy in pigs collectively referred to as prion diseases. A major feature of these diseases is that they are transmissible [33, 34].

Cerebrovascular diseases such as stroke cause acute degeneration of the CNS unlike the previously discussed chronic NDs. About 85% of stroke cases are of ischemic origin and have a slightly different etiology from the chronic ND. Interruption of blood supplies to the brain leads to a cascade of events that causes irreversible neuronal damage. Stroke is said to be the second leading cause of death in industrialized countries [35] and has been reported to lead to dementia in 25% of patients within three months after a stroke [36]. Interestingly, the deposition of both A*β* and APP in the cortical and subcortical brain areas of nondementia patients following stroke has been reported [37].

## 3. Ageing

Ageing has been defined as a complex physiological process involving both morphological and biochemical changes that occur progressively [1]. These changes include those of the CNS, the skin, the cardiovascular system, and hormonal and reproductive systems. Ageing remains to be the leading risk factor for NDs except for the familial forms which are found to affect individuals younger than 60 [1, 4, 5]. Human life expectancy has increased rapidly over the past decades especially in developed countries, and as the world populations get older, age-related NDs such as AD and PD have become more common [4, 38].

Although neuronal cell death is not programmed to occur at a particular age, cellular and molecular changes that occur with ageing interact with genes and environmental factors to determine which cells age successfully and which will suffer neurodegeneration [5]. Ageing is associated with mitochondrial dysfunction, increased free radical production and oxidative stress, microglia dysfunction, reduced efficiency of chaperones, reduced synaptic densities, blood-brain barrier disruption, and low levels of neurogenesis [5, 39, 40]. The accumulation of deletions of mitochondrial DNA in ageing alongside functional impairment of neurons results in age-related genetic changes in the substantia nigra [41]. The oxidative neuronal damage in the ageing brain is also associated with the decline in the production and function of antioxidant enzymes [41]. Oxidative damage to the mitochondrial DNA is believed to be caused by free radicals of mitochondrial origin. Furthermore, ageing comes with an alteration in the composition of the cell membrane fatty acids. The polyunsaturated fatty acids (PUFAs), like arachidonic acids, are abundant in ageing brain and are highly susceptible to oxidative damage like lipid peroxidation. Histopathological changes also occur with microglia morphology and activity during neuroinflammation in the aged brain. Moreover, aged microglia have shorter and less motile processes and larger soma sizes compared with microglia in younger animals [42]. Also, phagocytosis function of the microglia appears to be altered in the aged brain [43], and aged microglia exhibit increased expression of the phagosomal/lysosomal associated marker. There is increased levels of proinflammatory cytokines such as interleukin-6 (IL-6), IL-1*β*, and tumour necrosis factor (TNF), as well as altered levels of the anti-inflammatory cytokines such as IL-10 and transforming growth factor (TGF) *β*1 [39].

Progressive accumulation of iron in the brain and substantia nigra with age renders neuronal cells more susceptible to toxins [44]. The sirtuins, members of the histone deacetylase family of proteins, are also vital for cellular functions and play a role in ageing. Inhibition of sirtuin 2 (SIRT2) has been shown to rescue the toxicity of *α*-synuclein and modified inclusion morphology in animal models of PD, indicating a link between ageing and neurodegeneration [45]. The activity of proteasome, whose function is linked to the degradation of damaged or ubiquitinated proteins, is also known to be reduced with ageing. Consequently, an increase in abnormal deposition of cellular brain proteins continues with the ageing process [46]. The insulin/insulin-like growth factor 1 (IGF1) signaling pathway that serves as a lifespan, metabolism, and stress-resistance regulator has been found to be associated with neurodegeneration during the ageing process. As the optimal functioning of this pathway is key to maximizing longevity, a reduction of insulin signaling results in diabetes, reduced longevity, and prevalent protein aggregation-mediated toxicity [47]. There is also increasing evidence to suggest that cognitive dysfunction due to loss or decline of the cholinergic activity in the key areas of the brain is a normal biological process associated with ageing as well as some forms of progressive neurodegenerative disorders such as AD [48].

## 4. Common Pathophysiological Hallmark of Neuronal Cell Death

### 4.1. Protein Misfolding and Aggregation

For proteins to properly function in the biological system, they must maintain their three-dimensional conformation. The process by which polypeptide chains fold into this three-dimensional structure is termed protein folding. The correct three-dimensional structure, which is dependent on the sequence of amino acids, is very essential in the proper functioning of the protein. This intricate stepwise process can however go wrong leading to the adoption of abnormal configuration by the protein, a process called protein misfolding. Misfolded proteins lose their natural activity and in several cases become deleterious and are unable to return to their native conformation. These proteins expose hydrophobic terminals that are supposed to be buried in their core leading to the formation of insoluble aggregates. Since misfolded conformation can be generated spontaneously at low rate throughout life, their aggregates gradually accumulate as we age. These aggregates form distinct and observable structures in the brain which are generally known as amyloid deposits in the brain. Furthermore, some form of mutation increases the chance of misfolding in proteins which is the case in the genetic or familial case of NDs [22].

Though the mechanism is not clear, it has been proven that deposits of misfolded proteins are known to be neurotoxic and able to cause apoptosis in neuronal cells. There are however mechanisms that are able to inhibit aggregation of proteins and protect the CNS from such danger. Examples of these include the chaperones that are able to bind to misfolded protein and cause them to fold into their native structure and also the “ubiquitination” reaction. However, when these systems are unable to cope, aggregation takes place and the subsequent neuronal cell death becomes inevitable [46, 49].

As described in the preceding texts, two microscopic features resulting from the misfolding of proteins are observed in AD: extracellular amyloid plaques (senile plaque) consisting of amorphous extracellular deposits of A*β* and the NFTs of the hyperphosphorylated microtubule-associated tau protein [1, 22]. Amyloid deposits consist of aggregates containing 40 or 42 amino acid residues. Aggregates of 42 residue are more likely to form and are also overproduced when there is a genetic mutation [22]. In rare cases of early onset of PD which runs in some families, mutations in a synaptic protein called *α*-synuclein that was originally identified from smaller peptides isolated in amyloid-containing fractions of AD brains are observed [50, 51]. The *α*-synuclein proteins are synaptic proteins that are able to aggregate and form fibrils and are the major component of the Lewy body lesions, characteristic of PD as well as certain cases of AD and several other neurodegenerative conditions [52]. In the HD, mutation of huntingtin, a cytoplasmic protein, leads to its aggregation and forms inclusions in cell nucleus in the brain. These aggregations, alongside the interaction of mutated huntingtin protein with regulatory caspases, are believed to be component of the pathophysiology of HD [53]. In the case of ALS, it is the SOD that aggregates. There is evidence to suggest that the accumulation of SOD aggregate may lead to inflammation and neurotoxicity [54].

### 4.2. Oxidative Stress

Oxidative stress occurs when the body's antioxidant defense system can no longer cope with the neutralization of free radicals and/or reactive oxygen species (ROS) produced in the body. Free radicals have incomplete electron shells making them more chemically reactive than molecules with complete electron shells. They are formed in the course of normal cellular respiration and metabolism, especially under the influence of certain environmental chemicals and sunlight. When an oxygen molecule (O_2_) becomes electrically charged, it tries to steal electrons from neighboring molecules, causing damage to the DNA and other molecules. This damage may over time become irreversible, thereby leading to damage of cells and the body. The ROS are highly reactive molecules produced in the course of oxygen metabolism in the mitochondrial respiratory chain [55–59]. The term ROS when used implies superoxide radicals (O_2_^•−^), hydroxyl radicals (^•^OH), hydrogen peroxide (H_2_O_2_), and hydroperoxyl radicals (^•^HO_2_). Other ROS include reactive sulphur species and the reactive nitrogen species such as nitric oxide (NO) and peroxynitrite (O=N–O–O^−^). The brain with only 2% of the body mass but responsible for about 20% of the oxygen utilization is particularly susceptible to oxidative damage [1, 59]. Furthermore, the brain tissues are readily susceptible to lipid peroxidation reactions due to the high amount of PUFAs present in neuronal membranes. The result of this is the formation of cytotoxic aldehydes, such as malondialdehyde (MDA) and 4-hydroxynonenal [55–60]. Several studies have shown that cellular damage arising from free radicals and/or ROS is implicated in the etiology and pathophysiology of NDs and several other diseases [55–61].

### 4.3. Inflammation

Inflammation is part of the complex physiological response of the body to harmful stimuli or body injury by internal or external mechanical, chemical, or immunological agents. This protective mechanism can however become deleterious when it became excessive or out of control. In NDs, inflammation may result from aggregation of misfolded proteins, accumulation of abnormally modified cellular components, response to molecules released following neuronal injuries, and faulty regulation of inflammatory control mechanisms [62]. In AD, microglia are closely associated with neurons expressing A*β*. On the other hand, A*β* deposits in the brain are thought to be associated with an inflammatory response leading to increased levels of proinflammatory cytokines, complement components, and acute phase proteins [63]. Microglial cells act through a conserved group of receptors called pattern recognition receptors (PRRs). These PRRs include Toll-like receptors (TLRs) which bind conserved molecular motifs displayed by pathogen-associated molecular patterns (PAMPs) expressed by infectious agents or endogenous danger-associated molecular patterns (DAMPs) released from damaged tissues. Among DAMPs that have been identified are A*β*, *α*-synuclein, and microtubule-associated protein tau. Microglia and astrocytes can express various TLRs that once bound to their ligands, can stimulate the production of proinflammatory cytokines like TNF-*α* and IL-6 and chemokine such as IL-8 [64, 65].

As in AD, increased levels of proinflammatory cytokines are common in the blood, cerebrospinal fluid, and postmortem brain tissue of PD [66, 67]. Microglia induce the release of inflammatory cytokines and lead to activation of inflammation-mediating enzymes like matrix metalloproteinases (MMPs) following activation by *α*-synuclein [68, 69]. A component of inflammasomes, nucleotide-binding domain, and leucine-rich repeat family, pyrin domain containing 3 (NLRP3), is expressed by activated microglia. Inflammasomes stimulate several inflammatory processes including the maturation of IL-1*β* which has been reported to worsen AD and PD progression in animal models [70]. Nonsteroidal anti-inflammatory drugs have hence been proposed to have protective effects [71].

The neuroprotective role of microglia, cytokine, and other inflammation and immune mechanisms of the CNS are also well known [72]. For instance, microglia in the CNS has a role of clearing apoptotic cells and debris. There is also an evidence to show that microglia participates in the elimination of fibrillar A*β* through macropinocytosis [73]. However, chronic activation of microglia may lead to loss of this protective property causing an enhanced production of various cytokines which impair phagocytosis and other processes necessary for cell survival [74]. The new concept of “neuroinflammation” attempts to explain the involvement of inflammation in the pathology of AD and other NDs using available studies. However, the controversy as to whether inflammation is beneficial or detrimental to the CNS of ND patients is yet to be resolved. Consequently, “a common perspective, which implies that, under pathological conditions, inflammation may exert both detrimental and protective functions depending on local factors and the timing of immune activation and shutting-off systems” was proposed [72].

### 4.4. Neurotransmitter Level

A number of neurotransmitters and associated biochemical processes are involved in the pathology of NDs. These include ACh, DA, and MAO. Low level of neurotransmitters such as ACh and DA characterized the two major neurodegenerative diseases, AD and PD, respectively. A loss or downregulation of the neuronal nicotinic acetylcholine receptors (nAChRs) as well as loss of the central cholinergic neurons is associated with the pathogenesis of the AD brain [73].

The MAO catalyzes oxidative deamination of monoamines leading to the production of H_2_O_2_, aldehyde, ammonia, and amine, all of which have been found to be toxic at high concentrations and contribute to the pathology of NDs [74]. Accordingly, MAO-B inhibitors have been reported to provide mild symptomatic effects and to reduce the incidence of motor fluctuations in PD with fewer side effects. They also modify the disease state thus making them ideal candidates for the early treatment of the disease [75].

The DA is a neurotransmitter synthesized by mesencephalic neurons of the substantia nigra and ventral tegmental area and by hypothalamic neurons of the arcuate and periventricular nuclei [76]. Proper control of the DA levels and DA receptor interaction is important for normal functioning of the brain and several neurological and psychiatric disorders results from dysfunctional dopaminergic system. The dopaminergic pathways are implicated in several neurological and psychiatric diseases: for example, reduced levels of DA in PD, degeneration of selected DA neurons in HD, and dysfunctions of the dopaminergic system in ischemia and epilepsy [77]. Dopamine has also been shown to have either neuroprotective or neurotoxic effects in different physiological and pathological conditions [76, 78, 79].

## 5. Overview of the Current Pharmacotherapy

There is no cure for NDs and all current therapeutic interventions are aimed at managing the symptoms and improvement in the quality of life in patients. Therapies for AD, PD, and other NDs provide symptomatic improvement by several mechanisms such as restoring the levels of neurotransmitters or controlling the metabolism of neurotransmitters involved in the diseases [1, 12, 13, 21]. With the abounding evidences that the central cholinergic system plays a key role in the retrieval and storage of memory items in the CNS of mammals [25–27], some current therapeutic strategies are aimed at boosting the endogenous level of ACh in order to enhance the cholinergic deficits [80]. Some of these strategies include the use of muscarinic M_2_ autoreceptor inhibitors [81] and nicotinic agonists [82]; ACh releasers or donors [83]; ACh precursors [84]; and acetylcholinesterase (AChE) inhibitors which act by inhibiting the hydrolysis of ACh in the synaptic cleft thereby restoring the levels of the neurotransmitter [85, 86]. Among these strategies, enhancing cholinergic deficit using agents that can inhibit AChE has been the main focus of attention by researchers in recent years [27].

Most of the currently available US FDA-approved drugs for the treatment of cognitive impairment such as donepezil, tacrine, galanthamine, and rivastigmine elicit their memory enhancing effects mainly by inhibiting AChE [12, 87]. In 2003, memantine was approved for the management of moderate to severe cases of AD. It is a low affinity and noncompetitive* N*-methyl-D-aspartate (NMDA) receptor antagonist able to counteract neurotoxicity due to excitotoxicity of glutamate, the major excitatory neurotransmitter in the brain [1], without interfering with the physiological actions of glutamate necessary for memory and learning [12, 88]. Medications for PD include levodopa, a precursor of DA; DA receptor agonist like bromocriptine and ropinirole which can act on the dopamine receptors in the CNS; and anticholinergic drugs such as benztropine which acts to give symptomatic relief to tremors and muscle stiffness [89]. Also, drugs are given to take care of nonmotor symptoms of the disease. Examples include nortriptyline for depression related symptoms and the AChE inhibitors such as donepezil and rivastigmine [90].

## 6. Classical Examples of Neuroprotective Drugs of Plant-Origin and Their Mechanisms of Action(s)

### 6.1. Polyphenols

**Figure 1:**
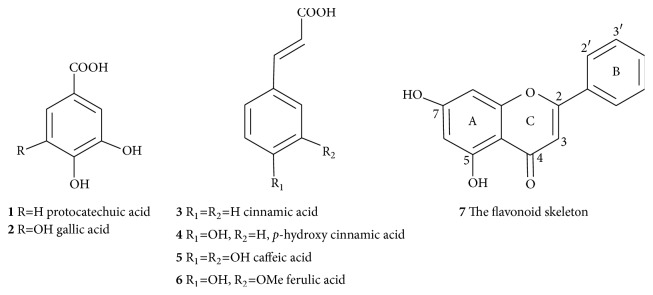
*Common phenolic compounds*. Structures of aromatic acids and the flavonoid skeleton are shown.

Polyphenols are diverse groups of plant secondary metabolites present in fruits, vegetables, legumes, cereals, and beverages like tea, coffee, and wine. One of the most common classes of polyphenols are the C6-C1 structural group of hydroxybenzoic acid derivatives such as protocatechuic (**1**) and gallic acid (**2**) that are derived from the shikimic acid biosynthesis pathway (Figure 1). The other diverse group of shikimic acid products of phenolics are the C6-C3 metabolites (phenylpropanoids) such as cinnamic (**3**),* p*-hydroxy cinnamic (**4**), and caffeic (**5**) and ferulic (**6**) acids which are abundant in many vegetables, fruits, and seeds (e.g., coffee beans).

The other major classes of natural polyphenolic compounds are flavonoids that constitute the C6-C3-C6 structural configurations (Figure 1). Structurally, they are composed of two six-member aromatic rings designated as A- and B-rings, joined together by three-carbon chain that may cyclise to form the third ring-C (**7**, Figure 1). Biosynthetically, the B-ring and linking three-carbon chain (C6-C3) are the phenylpropanoid skeleton that is derived from the shikimic acid pathway, while the other C-6 (A-ring) is an acetate pathway origin that bears the three hydroxyl groups spaced at* meta* positions from each other within the benzene ring. Depending on the position of attachment of the B-ring on the 3-carbon linking chain (at C-2 of the common flavonoids, C-3 of the isoflavonoids, and C-4 of the neoflavonoids), or the chemistry of the linking chain (e.g., presence/absence of a double bond, cyclisation, presence/absence of a ketone functional group), flavonoids are further divided into several classes such as flavones (apigenin, luteolin), flavonols (e.g., kaempferol and quercetin), flavanones, isoflavones, anthocyanidins, and chalcones. These polyphenolic compounds can be further esterified with each other or sugars to form polymeric macromolecules such as tannins. Although phenolic compounds are mainly derived from the shikimic acid and acetate pathways, other biosynthetic routes such as the terpenoids and alkaloids can also go through extensive aromatization and oxidation reactions to give rise to polyphenolic compounds.

One of the most important structural features of polyphenols is their potential to treat a diverse range of disease conditions including NDs and metabolic disorders (e.g., diabetes). It is also worth noting that complex diseases such as the NDs do not have one single pharmacological target and drugs working through a simple therapeutic principle (one drug → one-target → one disease) may not be effective, while drugs like polyphenols working through polypharmacology (multidrug → multitarget → multidisease/complex disease) or multifunctional (one drug → multitarget → multidisease/complex disease) principles have a far better chance to treat these diseases [91]. It is not surprising then that even reversing the neurotransmitter deficit in AD and PD does not offer a cure to NDs. Our decades of research have shown that polyphenolic compounds directly scavenge ROS and offer antioxidant effects that are of benefit to various disease conditions including NDs. The phenolic structural moiety plays vital role for antioxidant effect with the catecholic functional group being optimized for such activity and the gallic acid moiety even far better in inducing general antioxidant effects [92–103]. While the catechol structural moiety can also chelate metal ions, flavonoids especially those with the C-4 keto functional group possess another site of metal chelation with the 5-OH group, while those (e.g., flavonols) that bear a free hydroxyl group at C-3 position have even more pronounced metal chelation capability through the C-4 keto and C5/C-3 hydroxyl sites. Far beyond antioxidant effects [92–108], these polyphenols do also possess numerous other biological activities related to enzyme inhibition, gene expression, and signal transductions that attribute to numerous pharmacological effects including anti-inflammatory effects that play a significant role in NDs. Good examples of these polyphenols as well as nonphenolic compounds that have been shown to display neuroprotective effects are highlighted in the following sections.

#### 6.1.1. Phenolic Acids, Alcohols and Their Derivatives

**Figure 2:**
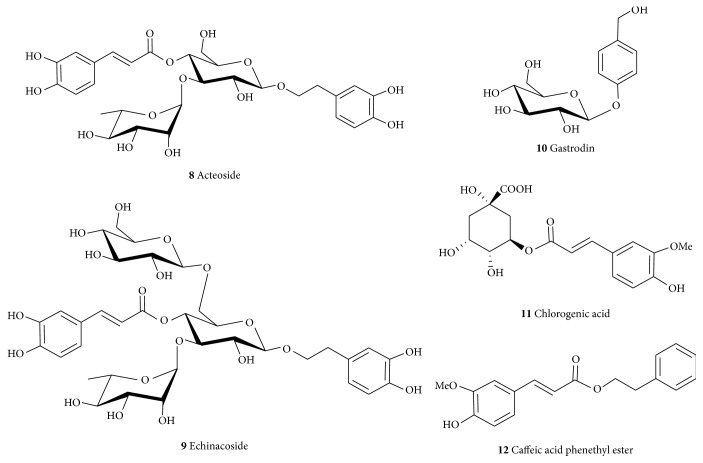
Structures of some common neuroprotective phenolic acid esters and glycosides.

Acteoside (**8**) and echinacoside (**9**) are glycosides (Figure 2) obtained from the dried juicy stem of* Cistanche deserticola* or* C. tubulosa* (Orobanchaceae) as well as many other plants. They have been shown to have neuroprotective effects on dopaminergic neurons of substantia nigra in a chronically intoxicated MPTP (1-methyl-4-phenyl-l,2,3,6-tetrahydropyridine) mice model of PD [109]. Acteoside (**8**) showed neuroprotective effects against the rotenone-induced damage to SH-SY5Y cells [110] and attenuated the lipopolysaccharide- (LPS-) induced release of NO in RAW 264.7 cells through inhibition of NF-*κ*B and activator protein-1 (AP-1) [111]. Its neuroprotective effects in the MPTP models of PD revealed that it improved behavioral deficits in C57BL/6 mice and increased the dopaminergic neurons and content of DA [112]. The neuroprotective assessment of echinacoside (**9**) showed that it prevents the 6-hydroxydopamine- (6-OHDA-) induced extracellular loss of monoamine neurotransmitters, such as 3,4-dihydroxyphenylacetic acid (DOPAC) and homovanillic acid in rat striatum [113]. In the MPTP-intoxicated mice model,** 9** reduced behavioral deficits, cell death, and MPP+-induced activation of caspase-3 and caspase-8 in cerebellar granule neurons as well as increased tyrosine hydroxylase (TH) expression and the level of striatal DA and its metabolite [114]. The compound (**9**) is active orally as an inhibitor of apoptosis and inducer of neurotrophic factors [115]. There is no doubt that the active principles of these compounds (**8**,** 9**) are the catechol functional moiety presented in the 3,4-dihydroxyphenyl ethanol and caffeic acid moieties in their structures. The extended double bond in the caffeic acid further stabilized the free radical generation/and or scavenging making it more active as antioxidant and related pharmacological effects. The neuroprotective effect of caffeic acid as a free acid and in its ester form such as chlorogenic acid (**11**) and caffeic acid phenethyl ester (**12**) has recently been reviewed [108]. Besides the common global antioxidant effects through direct scavenging of ROS and metal chelation, caffeic acid and its derivatives display specific anti-inflammatory mechanisms in the brain along with inhibition of key APP processing enzyme, *β*-secretase (BACE-1) [116]. Treatment of neuronal cells* in vitro* with** 11** has been shown to reverse the A*β*-induced neurotoxicity [117] while** 12** enhances the expression antioxidant proteins such as HO-1 (heme oxygenase 1) and the Nrf2 (nuclear factor (erythroid-derived 2)-like 2) in microglia cells both* in vitro* and* in vivo* [118]. We have also shown that gallic acid (**2**) and related natural products display neuroprotective effects both* in vitro* and* in vivo* under a number of stress conditions [119–122]. In addition to the above-mentioned plethora of pharmacological effects, modulation of the human microRNA-17-3p expression has been shown as the possible mechanism of action for the* in vitro* neuroprotective effect of gallic acid derivatives [123]. Hence, compounds bearing the catechol functional groups such as gallate or caffeic acid have been proposed to offer benefit in AD and related NDs [108].

Gastrodin (**10**) is another example of simple derivatives of the C6-C1 shikimic acid biosynthetic products that may offer benefit in NDs. It has been obtained from* Gastrodia elata* (Orchidaceae) among other plants [124]. Gastrodin (**10**) showed neuroprotective effects in the subchronic MPTP mouse PD model by ameliorating bradykinesia and motor impairment [125]. It also protected dopaminergic neurons against neurotoxicity through regulating free radicals, Bax/Bcl-2 mRNA, and caspase-3 and cleaved poly-ADP-ribose polymerase (PARP) in SH-SY5Y cells stressed with MPP+ [125]. The compound also prevents neuronal apoptosis by attenuating oxidative stress and inhibits the levels of neurotoxic proinflammatory mediators and cytokines including iNOS, COX-2, TNF-*α*, and IL-1*β* [126].

#### 6.1.2. Curcuminoids and Resveratrol

**Figure 3:**
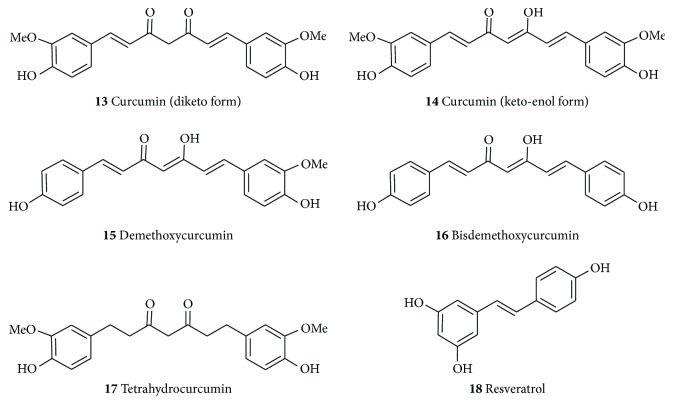
Structures of curcuminoids and resveratrol.

The rhizomes of turmeric (*Curcuma longa* L., Zingiberaceae) and related species have long been known for their use as spices and medicinal properties with their characteristic yellow colour attributed to the principal pharmacologically active principle, curcumin (**13**, Figure 3). Curcumin can exist as a diketo (**13**) or keto-enol form (**14**) while other structurally related derivatives isolated from the turmeric in significant amount include demethoxycurcumin (**15**) and bisdemethoxycurcumin (**16**). The two aromatic phenol rings which are connected by two sets of *α*,*β*-unsaturated carbonyl groups are good Michael acceptor and interact with a range of biologically reactive species including glutathione and other nucleophiles, while the aryl methoxyl groups at the ortho position to the hydroxyl moiety as well as the conjugated *β*-diketone moieties of curcumin are important structural features for the various pharmacological activities of these compounds [127]. The range of biological activities for these compounds collectively, called the curcuminoids, has been reviewed in the various literature (e.g., [128]) and numerous synthetic analogues have also been evaluated in recent years. The presentation herein is thus limited to highlight that curcumin is one classical example of natural products that may be employed as a neuroprotective agent through multifunctional mechanism of actions.

Curcuminoids as well as the crude extracts of* C. longa* have been extensively studied for their neuroprotective effects [129–131]. They prevented MPTP mediated loss of TH-positive neurons and depletion of DA in inflammation-mediated neurodegeneration of dopaminergic neurons of C57BL/6 mice in the acute MPTP model. They also mitigated cytokines, generation of NO, and the expression of protein inflammatory markers as well as improved motor deficits produced by the MPTP [129]. Curcumin (**13**) has also been shown to have neuroprotective effect in a 6-OHDA-induced hemiparkinsonian mice model. It decreased the 6-OHDA-induced loss of striatal TH fibers and nigral TH-immunoreactive neurons indicating that the neuroprotective effects may be due to its anti-inflammatory properties, or direct protection on nigral DA neurons [131]. Curcumin was also found to ameliorate the A53T *α*-synuclein-induced SH-SY5Y cell death [132] and decrease *α*-synuclein-induced intracellular ROS generation and inhibit caspase-3 activation in the SH-SY5Y cells [133]. It also protects dopaminergic neurons from apoptosis in an MPTP mouse model of PD and ameliorated the loss of dopaminergic axons. It prevents the degeneration of nigrostriatal neurons by inhibiting the dysfunction of mitochondria through abolishing the hyperphosphorylation of c-Jun N-terminal kinase (JNK) induced by MPTP [130]. Several studies have reported that curcumin has antioxidant, anti-inflammatory, and antitumor activity. In* in vitro* studies, curcumin inhibited the activities of *β*-secretase, AChE, as well as A*β* aggregation, and A*β*-induced inflammation. Studies in animal models of AD also indicate a direct effect of curcumin in decreasing the amyloid pathology of AD, and oral administration resulted in the inhibition of A*β* deposition, A*β* oligomerization, and tau phosphorylation in the brains of AD animal models and improvements in behavioral impairment [134–140].

In comparing curcumin and its more stable metabolite, tetrahydrocurcumin (**17**, Figure 3), it was observed that the dienone bridge present in curcumin, but not in TC, is important in reducing plaque deposition and protein oxidation in an AD's model even though** 17** reduced neuroinflammation and soluble A*β*, an effect that can be attributed to limiting JNK-mediated transcription [135]. An alternative mechanism of curcumin in reducing A*β* aggregation or oxidative neurotoxicity is through metal chelation. It interacts with copper and iron thereby offering a net protective effect against A*β* toxicity and suppressing inflammatory damage by preventing the induction of NF-*κ*B [141].

Resveratrol (**18**) is a natural stilbene polyphenol found in red grapes, peanuts, and tea. It is found in the skin of red grape and can be extracted by fermentation of the skin. It is also found in high concentration in the oriental plant,* Polygonum cuspidatum* (Polygonaceae), used to treat fevers, hyperlipidemia, atherosclerosis, and inflammation [142]. Though the full understanding of its neuroprotective mechanism is yet to be outlined,* in vitro* and* in vivo* studies have shown that** 18 **can elicit a wide range of beneficial effects on NDs. Among the several possible mechanisms by which** 18** is thought to act, its antioxidant activity seems to be most important [143]. Given that oxidative stress is implicated in the pathologies of AD, PD, HD, prion disease, cerebral ischemia, ALS, and other NDs [55–61], a great deal of emphasis has been given to study the antioxidant effect of** 18** in the respective NDs models. Furthermore,** 18** ameliorates the deleterious effects triggered by oxidative stress through activation of SIRT1 and vitagenes which can modulate ROS production [144]. Furthermore, it acts by activating SIRT1 and the PGC1*α* pathway. Both pathways are known to lead to improved mitochondrial function and efficiency [143]. Resveratrol also inhibits A*β* formation through sirtuin-dependent activation as potentiation of the activity of SIRT1 has been noted [144]. The SIRT1 can deacetylate tau protein at multiple residue* in vitro* exposing the lysine residue to ubiquitin ligases leading to the marking of the tau protein for proteosomal degradation [145]. Allosteric modulation of resveratrol (**18**) on SIRT1 activates the deacetylation of p53, a protein that induces indirect phosphorylation of tau which has been reported to be unregulated in the superior temporal gyrus in AD [146].

Similarly in PD, the antioxidant properties of resveratrol (**18**) are believed to play major role in its neuroprotective effect on dopaminergic neurons [147]. Resveratrol also via inducing the activation and expression of SIRT1 offers protection against pathological *α*-synuclein aggregation [143]. Another possible mechanism by which** 18** can decrease *α*-synuclein protein expression in cellular model of PD is through the downregulation and partial inhibition of GSK-3*β* (glycogen synthase kinase 3 beta) which has been shown to protect the dopaminergic neurons from various stress-induced injuries. Resveratrol (**18**) can also prevent calcium elevation caused by repeated and persistent entry of calcium into the cells as a result of intracellular calcium oscillation following monoamine induced metabolism of DA and the production of H_2_O_2_ in PD patients [58, 143]. On the basis of all the available data so far, resveratrol (**18**) has been widely reported as a useful natural potential therapeutic agent in managing NDs [148–154].

#### 6.1.3. Flavonoids

**Figure 4:**
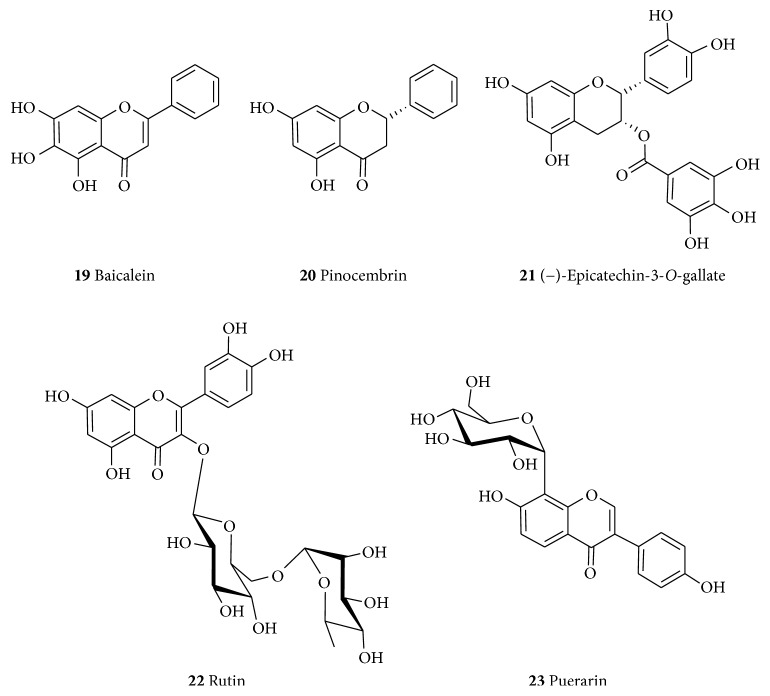
Examples of flavonoids with neuroprotective effects.

Flavonoids are one of by far the best studied groups of compounds for their neuroprotective effects and a selected few are presented here as classical examples of their representative structural class. Baicalein (**19**, Figure 4) belongs to the flavone class of flavonoids that has been isolated as the principal component of* Scutellaria baicalensis* (Labiatae). Structurally, this compound has trihydroxy substitution in the A-ring that mirrors that of the gallate (**2**) bioactive structural moiety. The compound has been evaluated for its neuroprotective effect in the 6-OHDA-induced cellular and animal models of parkinsonism. It promoted neurite outgrowth in PC12 cells and attenuated the 6-OHDA-induced cell apoptosis in the SH-SY5Y cells. It also attenuated muscle tremor in 6-OHDA-lesioned rats and mitigated against astroglial response and increased TH-positive neurons in substantia nigra [155]. The effect of baicalein (**19**) on rotenone-induced neurotoxicity in PC12 cells was also evaluated. It inhibits the accumulation of ROS, suppressed rotenone-induced apoptosis and production of ROS, promotes mitochondrial active respiration, and prevents the rotenone-induced deficiency of ATP and swelling of isolated brain mitochondria [156]. Baicalein (**19**) also protects the SH-SY5Y cells from *α*-synuclein oligomer-induced toxicity [157] and against the MPTP-induced fall of TH-positive neurons in the substantia nigra as well as preventing an MPTP-induced decrease in DA levels [158]. It has also been shown to improve impaired spontaneous motor activity and rotarod performance induced by MPTP in C57BL/6 mice [158]; modulate the balance between glutamate and gamma amino butyric acid [159]; increase the counts of dopaminergic neurons [103]; and enhance the levels of DA and 5-hydroxytryptamine in the striatum [158]. Baicalein (**19**) also inhibits the following: cytochrome oxidase subunit I (CO-I) mRNA expression in the subthalamic nucleus [115], the oxidative stress and the astroglial response [160], the oligomerization of *α*-synuclein in cell-free and cellular systems, *α*-synuclein fibrillation in cell-free systems, and the formation of *α*-synuclein oligomers in HeLa and SH-SY5Y cells [157]. On the other hand, the ethanolic extract of* S. baicalensis* was shown to decrease the LPS-induced expression of inducible nitric oxide synthase (iNOS), NO, cyclooxygenase-2 (COX-2), and prostaglandin E2 levels in BV-2 and RAW264.7 cells [160]. The neuroprotective effect of** 19** through antioxidant and mitochondrial mechanisms hs also been reviewed [161].

Flavonoids have also generally been implicated in the management of AD [162–164]. Hence, baicalein (**19**) has been reported to prevent A*β*-induced impairments in hippocampal LTP through activation of Akt phosphorylation and memory deficits in AD model. It also inhibits 12/15-lipoxygenases and GSK3*β* activity, reduces BACE1 and A*β* production, prevents tau phosphorylation, and restores spine density and LTP in AD model [165, 166]. In addition to inhibiting A*β*-induced depolarization,** 19** also functions as an antagonist of AMPA (*α*-amino-3-hydroxy-5-methyl-4-isoxazolepropionic acid) and NMDA receptors and improves neurocognition [167, 168]. It also attenuates neurological deficits and preserves blood-brain barrier integrity in a rat model of intracerebral hemorrhage and inhibits AChE [169–172]. Recently, cocrystals of baicalein with isoniazid, isonicotinamide, caffeine, and theophylline were developed with BaiCaf having superior powder dissolution and pharmacokinetic behaviors [173].

Pinocembrin (**20**) is a flavonoid (favanone subclass) isolated from several plants including the* Pinus* heartwood,* Eucalyptus, Populus, Euphorbia*, and* Sparattosperma leucanthum* [174]. The neuroprotective effects of** 20** against cerebral ischemic injury have been reported with a wide therapeutic window that has been shown to be attributed to its antiexcitotoxic effects [175]. It has been shown that** 20 **alleviates cerebral ischemic injury induced by occlusion of the middle cerebral artery in rats [176, 177] as well as enhanced cognition by protecting cerebral mitochondria structure and function against chronic cerebral hypoperfusion in rats [178]. Other mechanisms of action include increasing ADP : oxygen ratio, glutathione, state 3 respiration state, neuronal survival rates, and oxidative phosphorylation rate in NADH/FADH_2_ and decreasing LDH release, ROS, NO, neuronal NOS (nNOS), iNOS, and 4 respiration state (V4) in NADH [177]. It also enhances ATP content in brain mitochondria in the SH-SY5Y cells [175, 179]. Furthermore, it decreased brain edema, improved cerebral blood flow, and increased the viability and mitochondrial membrane potential of cultured rat cerebral microvascular endothelial cells [180]. Pinocembrin (**20**) also possesses anti-inflammatory [181, 182] and antioxidant activities [183, 184]. Pinocembrin improves cognition and protects the neurovascular unit in Alzheimer-related deficits and inhibits A*β*-induced neurotoxicity through Nrf2/HO-1 pathway in the SH-SY5Y cells [185–187].

The consumption of tea has been shown to be inversely correlated with the incidence of dementia, AD, and PD due primarily to its main catechin polyphenol constituent (−)-epigallocatechin-3-*O*-gallate (**21**) among others. The (−)-epigallocatechin-3-*O*-gallate (**21**) has been reported to show neuroprotective effects in many cellular and animal models of neurological disorders. In addition to the known antioxidant activity of catechins, the modulation of signal transduction pathways, cell survival/death genes, and mitochondrial function all contribute significantly to the induction of neuron viability [188]. Recent studies have linked the biological activities of catechins to their ability to modulate various protein kinase signaling pathways in addition to their antioxidant/radical-scavenging potential [188, 189]. Hence,** 21** has been shown to improve cognitive decline associated with age as well as protect against cerebral ischemia/reperfusion injuries [190] and brain inflammation and neuronal damage in experimental autoimmune encephalomyelitis [191]. It also prevented striatal DA depletion and substantia nigra dopaminergic neuron loss in the MPTP PD model [192]. It reduces cerebral amyloidosis in AD's transgenic mice [193] and prevented neuronal cell death due to 6-OHDA, 1-methyl-4-phenylpyridinium and A*β* [194, 195]. It boosts cellular protein stability and prevents cell damage and cell death by modulating macroautophagy [196]. Other complex mechanisms of action that may explain its beneficial neuroprotective effects have also been reported [197].

The flavonol glycoside, rutin (**22**), its aglycone quercetin, and a related glycoside isoquercetin (quercetin 3-*O*-glucoside) have been shown to possess neuroprotective effects on glutamate-induced oxidative injury in the HT22 hippocampal cells [198, 199]. Rutin (**22**) reportedly reduces the levels of ROS, MDA, NLRP3 (NLR family pyrin domain containing 3), ASC (apoptosis-associated speck-like protein containing a CARD), caspase-1, IL-1*β*, and IL-18 and attenuates histologic alteration as well as improving locomotion recovery [200]. Quercetin also dose-dependently reduced the A*β*- (A*β*_(1–42)_-) induced paralysis in* Caenorhabditis elegans *by decreasing the amount of aggregated proteins [201] and antagonized the high glucose-induced damage of Schwann cells by inducing autophagy [202]. Rutin (**22**) attenuates the isoflurane-induced neuroapoptosis by modulating JNK (c-June-N-terminal kinase) and p38 MAPK (mitogen-activated protein kinase) pathways in the hippocampi of neonatal rats [203]. It also protects against neuronal damage* in vitro* and ameliorates doxorubicin-induced memory deficits* in vivo* in Wistar rats [204]. Rutin (**22**) has also shown to inhibit protein aggregate formation, depolymerize aggregates, and reduce neurotoxicity [205]. Details of the mechanism of action of rutin and related compounds have been outlined in our previous review communication [199]. 

Puerarin (**23**) is an example of the isoflavonoid subclass of flavonoid compounds that has been obtained from* Pueraria lobata* (Leguminosae) [206, 207]. It has been observed to upregulate the phosphorylation of Akt in MPP+-induced cytotoxicity in SH-SY5Y cells and inhibits MPP+-induced nuclear translocation of p53, expression of Bax, and caspase-3-dependent programmed cell death [208]. Puerarin (**22**) was also reported to prevent the dysfunction of the proteasomal system thus preventing the accumulation of conjugated proteins [209]. It was shown to suppress the LPS-induced release of iNOS and phosphorylation of MAPKs in N9 cells [210] and inhibits the MPP+-induced phosphorylation of JNK [211]. In 6-OHDA-lesioned rats,** 23** significantly increased the protein expression of DJ-1 and SOD-2 in the substantia nigra [212] and increased the expression of the glial cell-derived neurotrophic factor in the striatum of rats administered with 6-OHDA [213]. Other studies have also shown the neuroprotective effects of** 23** in AD [214, 215]. Puerarin also suppresses the A*β*_(1–42)_-induced primary cortical neuron death [216] thus protecting against A*β*_(1–42)_-induced learning and memory impairments in mice [217]. It also attenuates myocardial hypoxia/reoxygenation injury by inhibiting autophagy via the Akt signaling pathway and inhibits oxidative stress in STZ-induced SAD mice [218, 219]. Overall, numerous reports from our laboratories and others have shown the neuroprotective effects of various classes of flavonoids (e.g., [220, 221]). Extensive literature review articles in the field are also available [220–226].

#### 6.1.4. Other Phenolic Compounds

**Figure 5:**
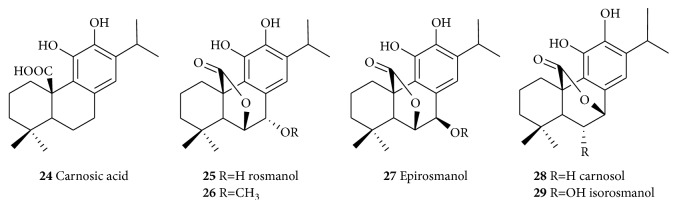
Bioactive phenolic compounds from Rosemary with potential neuroprotective effects.

Numerous phenolic compounds that are not of the acetate/shikimate pathway origin but with promising neuroprotective effects have been reported [227]. Of significance are the diterpenoids* Rosmarinus officinalis* (**24**–**29**, Figure 5) which have significant antioxidant, anti-inflammatory, antiamyloid, and anti-AChE activities [228]. Carnosic acid (**24**) from* R. officinalis* was reported to have neuroprotective effects on cyanide-induced brain damage in cultured rodent [229] while the protective effects of carnosol (**28**)* in vitro* on rotenone-induced neurotoxicity in cultured dopaminergic cells have also been reported [230]. The antioxidant potential of carnosic acid (**24**) and carnosol (**28**) has been well documented and their detailed neuroprotective mechanism have been reviewed recently [228]. For example, carnosic acid protects neuronal cells from ischemic injury by scavenging ROS [231] while carnosol inhibits the CU^2+^-induced LDL oxidation [232] and chelate Fe^2+^ [233]. On inflammation, carnosol (**28**) inhibits the adhesion of TNF-*α*-activated monocytes to endothelial cells and suppresses the expression of intercellular adhesion molecule at the transcriptional level [234]. It also inhibits the TNF-*α*-induced signaling pathways through inhibitor of NF*κ*-B (IKK-*β*) activity as well as the upregulation of HO-1 expression [235]. Both carnosic acid (**24**) and carnosol (**28**) antagonized intracellular Ca^2+^ mobilization induced by a chemotactic stimulus, coupled with inhibition of ROS generation [236]. Carnosic acid (**24**) also suppressed A*β*42 secretion [237, 238] and inhibited AChE [239, 240] and its therapeutic potential for AD both* in vitro* and* in vivo* is well documented [241, 242].

### 6.2. Alkaloids

**Figure 6:**
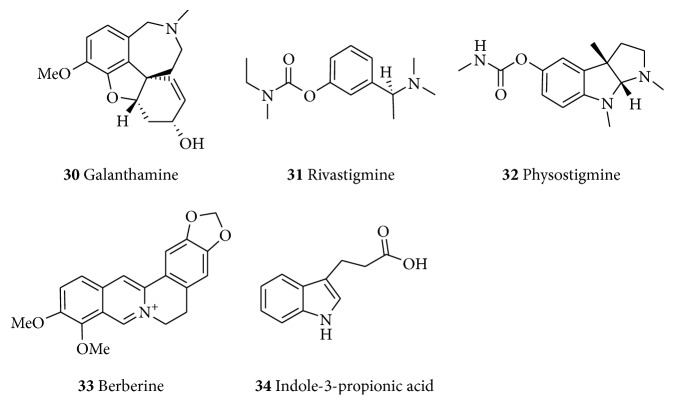
Examples of alkaloids with neuroprotective effects.

Galantamine (**30**, Figure 6) is an alkaloid originally isolated from* Galanthus woronowii* (Amaryllidaceae) and related species including* G. caucasicus* as well as from the related genera like* Narcissus*,* Leucojum (Leucojum aestivum),* and* Lycoris (Lycoris radiate)* [243]; but now it can also be obtained synthetically. With the trademark of Razadyne or Nivalin, galantamine (**30**) is an approved drug for the treatment of mild to moderate dementia in AD. Much work on its identification and properties was done by Mashkovsky and Kruglikova-Lvova in the 1950s. Before the FDA approval for its use for the management of AD in the US, the compound has been used for decades in Eastern Europe and Russia for various indications such as treatment of poliomyelitis, myasthenia, and myopathy. It had also found clinical use as anesthetics to reverse neuromuscular paralysis by tubocurarine-like muscle relaxants [244]. Galantamine (**30**) acts as a selective, competitive, and reversible inhibitor of AChE with very little butyrylcholinesterase (BuChE) inhibitory activity [244]. The selectivity is in the range of 10–50-fold for AChE than for BuChE [245]. As a competitive inhibitor, it competes with ACh at the AChE binding site, and since this is dependent on ACh concentration, it may be less likely to bind to the enzymatic site in areas with high ACh concentration [246]. It is also proposed to be an allosteric ligand at nAChR thereby inducing its modulatory effect leading to enhanced effect of ACh and cholinergic transmission [86]. The neuromodulatory activity at nicotinic receptors in addition to its effect on cholinergic transmission affects other neurotransmitter systems such as monoamines, glutamate, and *γ*-aminobutyric acid (GABA). These effects may improve cognitive dysfunction and psychiatric illness in schizophrenia, major depression, bipolar disorder, and alcohol abuse [247]. Galanthamine (**30**) showed protective effects on NO-mediated glutamate neurotoxicity when tested on primary cultures from the cerebral cortex of fetal rats. It also prevented the glutamate- and ionomycin-induced neurotoxicity [248]. The neuroprotective effect against both acute and moderate glutamate treatments was mediated through nAChRs *α*4- and *α*7 and phosphatidylinositol 3-kinase-Akt pathway [249, 250]. Galanthamine appears less tolerated when compared to other approved drugs but better toleration was noted when gradually titrated over more than three months [86].

Rivastigmine (trade name Exelon,** 31**) is another approved drug of natural origin for the treatment of AD. It is a semisynthetic derivative of physostigmine (**32**) obtained from* Physostigma venenosum* (Fabaceae). Also called Calabar bean,* P. venenosum* was used for ordeal poisoning by locals in the 19th century [251]. Rivastigmine, a powerful carbamate inhibitor, inhibits both BuChE and AChE by covalently binding to the esteratic part of the active site of the AChE in a slow-reversible manner. In addition to AD, rivastigmine is also indicated for the management of Lewy body dementia and PD dementia [252]. Rivastigmine (**31**) which was initially formulated in tablet form is now available as transdermal drug delivery systems (drug “patches”) which has the advantage of providing controlled and continuous delivery of drugs through the skin, minimizing the processing of the drug in the liver, stomach, and intestines as well as improving compliance [85]. It is also able to reduce the side effects associated with the use of the drug [86].

Berberine (**33**) is an isoquinoline alkaloid of the protoberberine type isolated from numerous plants with multiple therapeutic implications. It shows neuroprotective effect in various animal models of CNS-related disorders [253, 254]. Berberine (**33**) has D2 dopamine receptor antagonist and D1 dopamine receptor agonist effects [255]. It reverses the A*β*_(1–40)_-induced memory [256], reduces A*β*-production by modulating APP processing in human neuroglioma H4 cells [257], and inhibits AChE [258, 259]. It also reversed the NMDA-induced excitotoxicity [260] and reduced D1 DA and NMDA receptor bindings in the cortex [261]. Given that berberine (**33**) is a multifunctional compound with broad range of pharmacological effects ranging from anti-inflammatory and antioxidant to neuroprotection in a variety of models [262], it has been the subject of intense medicinal chemistry researches that aim to synthesize a better novel lead derivative compound [263]. The rather simple compound, indole-3-propionic acid (**34**) that can be obtained by deamination of the amino acid tryptophan, is a further example of neuroprotective natural products that can be obtained through a simple biosynthetic route [264]. Compound** 34**, isolated from* Clostridium sporogenes* (Gram-positive bacteria), has been reported as a potent antioxidant that attenuates neuronal damage and oxidative stress in the CNS [265]. It has also been detected in the plasma and cerebrospinal fluid of humans [266] where it offers complete protection to primary neurons and neuroblastoma cells against oxidative damage and death caused by exposure to A*β* [267]. This suggests that** 34** may form part of the body's natural defense against neuronal injuries due to free radicals [267] which is the basis for the development of some probiotic drugs such as OXIGON™ for the management of NDs.

### 6.3. Terpenoids

#### 6.3.1. Triterpenoids

**Figure 7:**
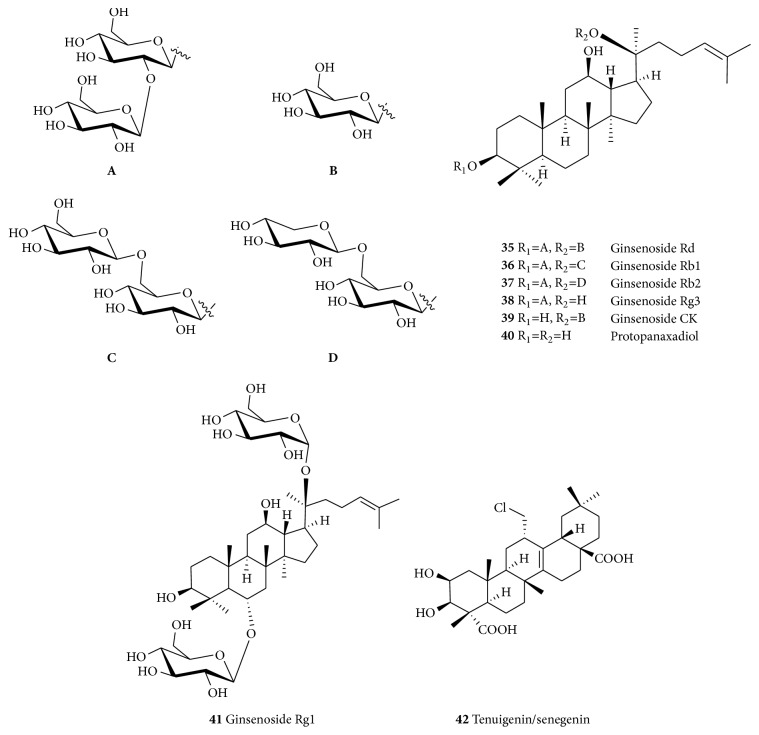
Structures of ginsenosides and tenuigenin.

*Ginsenosides* (**35**–**41**, Figure 7) are good examples of neuroprotective triterpenoids obtained from the roots and rhizomes of* P. ginseng *and* P. notoginseng* (Araliaceae) [268]. The aqueous extract of* P. ginseng* has been evaluated for its neuroprotective effects against the MPP+-induced cytotoxicity in SH-SY5Y human neuroblastoma cells [129]. It was observed that the extract reduced the overproduction of reactive ROS, release of cytochrome c, and activation of caspase-3 and elevated Bax/Bcl-2 ratio, thereby increasing the cell survival. Ginsenoside Rg1 (**41**) was reported to decrease the cytotoxicity induced by H_2_O_2_ in PC12 cells thus explaining its antioxidant activity. It also protects cells from injury induced by H_2_O_2_ by downregulating the ERK1/2 (extracellular signal-regulated kinases 1/2) as well as decreasing the activation of the NF-*κ*B signaling pathway [269]. Other studies showed that ginsenoside Rg1 (**41**) renews an iron-induced reduction in mitochondrial transmembrane potential, inhibits the 6-OHDA-induced upregulation of an iron importer protein divalent metal transporter 1 with iron responsive element (DMT1-IRE), inhibits iron regulatory proteins, thereby downregulating the DMT1-IRE expression [270], inhibits the MPP+-induced upregulation of DMT1-IRE [271], attenuates the MPTP-induced elevated iron levels, decreases the expression of DMT1, and increased ferroportin-1 expression [272].

Ginsenoside Rg1 (**41**) was found to have a neuroprotective effect in dopaminergic neurons through IGF1 receptor signaling pathway in a 6-OHDA-induced nigrostriatal injury model of PD [273]. It also has protective effects on dopaminergic neurons in ovariectomized female SD rats injected intracerebroventricularly with 6-OHDA [274]. Compound** 41** also has anti-inflammatory effect: it inhibits proinflammatory markers such as iNOS, NO, and TNF-*α* and expression of ionized calcium binding adaptor molecule 1 in both the cerebral cortex and hippocampus of mice as well as suppressing effect on the downstream inflammatory markers [275]. Saponins obtained from* P. notoginseng *also showed potent neuroprotective effect on MPP+-induced toxicity to PC12 cells and Kunming mice [276, 277].

Ginsenoside-Rg1 (**41**) has neuroprotective effect on cerebral ischemia/reperfusion injury in rats by downregulating protease-activated receptor-1 expression [278]. It can reduce neuronal death induced by hypoxic–ischemic insults, an effect probably mediated by the activation of glucocorticoid receptors, and by the inhibition of calcium influx through NMDA receptors and L-type voltage-dependent Ca^2+^ channels and the resultant reduction of intracellular free Ca^2+^ [279]. It also enhances neurite outgrowth and protects against neurotoxicity induced by A*β* through a mechanism involving Akt and ERK 1/2 signaling [280]. This compound (**41**) is also shown to be capable of helping Schwann cells recover from the oxidative insult induced by H_2_O_2_. It upregulates the level of SOD, GSH, and CAT and decreased the level of MDA in Schwann cells treated with H_2_O_2_ both* in vitro* and* in vivo* (sciatic crush injury model in rats). It has also shown to inhibit the proapoptotic effect of H_2_O_2_, as well as the detrimental effect of H_2_O_2_ on cell number and cell viability. Furthermore, it increases the mRNA levels, protein levels, and secretion of nerve growth factor and brain-derived neurotrophic factor in Schwann cells after incubation with H_2_O_2_ [281]. Ginsenoside Rg1 (**41**) as well as** 38** and others have been shown to reduce the amount of A*β* detected in the brains of animals after single oral doses [280]. Review articles on the effects of ginseng extracts and isolated ginsenosides along with the aglycones relevant to cognition in humans are also available [282, 283].

*Tenuigenin* (**42**) obtained from the dried root of* Polygala tenuifolia *(Polygalaceae) [284] is another example of a triterpenoid neuroprotective agent. Evaluation in the 6-OHDA-induced cytotoxicity in SH-SY5Y cells showed that tenuigenin (**42**) increased cell viability and reduced cell death [284]. It also protects against the 6-OHDA-induced damage of the mitochondrial membrane and increased glutathione and SOD expression [284]. It has also been shown to downregulate the caspase-3 activity at the translational level and upregulate the expression of TH in 6-OHDA thus indicating neuroprotective effects on dopaminergic neurons through antiapoptotic and antioxidant mechanisms [284]. It also showed neuroprotective effect on neuroinflammation induced by the LPS in adult male SD rat by significantly improving the level of DA in the striatum and preventing the LPS-induced upregulation of cytokines [285].

Tenuigenin (**42**) exerts an anti-inflammatory effect by downregulating the release of NO, MMP-9, and cytokines. It can decrease the release of NO from the LPS-activated rat microglia in a dose-dependent manner and directly scavenge the NO radical. It has also been shown to decrease the secretion and mRNA levels of MMP-9 and proinflammatory cytokines (TNF-*α*/IL-1*β*) in activated microglia as well as inhibit the secretion of MMP-2 [286]. It has also been reported that tenuigenin (**42**) inhibits the LPS-induced TNF-*α*, IL-1*β*, IL-6, and PGE_2_ production while the expression of Nrf2 and HO-1 was shown to be upregulated in a dose-dependent manner. In* in vivo *studies, it relieved the LPS-induced memory deficit in the Morris water maze and passive avoidance tests. This suggests the anti-inflammatory mechanism of neuroprotection through activation of the Nrf2-mediated HO-1 signaling pathway [287].

#### 6.3.2. Diterpenoids

**Figure 8:**
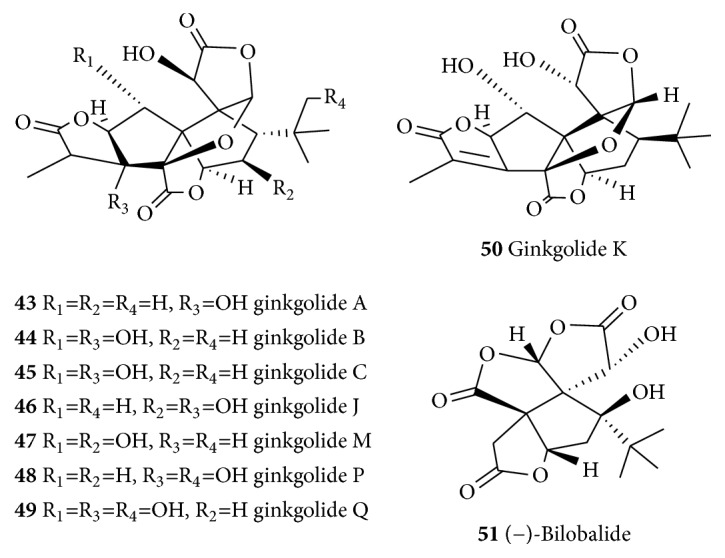
Structures of ginkgolides and related compounds.

In addition to the polyphenolic diterpenoids of Rosemary that have already been discussed in the preceding sections, several other diterpenes have been shown promising effect as neuroprotective agents. Ginkgolides (**43**–**49**, Figure 8) are a group of diterpenoids isolated from* Ginkgo biloba* (Ginkgoaceae), an ancient Chinese tree known for its health prompting effects. There have been several reports on the neuroprotective properties of* G. biloba* [288–290]. Hence, the extract of* G. biloba* has been reported to offer neuroprotection in the MPTP-induced nigrostriatal dopaminergic toxicity in C57 mice [291]. The various ginkgolides isolated from the plant have also been linked with neuroprotection. Ginkgolides protect PC12 cells against hypoxia-induced injury by p42/p44 MAPK pathway-dependent upregulation of HIF-1*α* (hypoxia-inducible factor 1*α*) expression and HIF-1-DNA binding activity [292]. The neuroprotective effect of ginkgolide K (**50**), for example, on glutamate-induced cytotoxicity in PC12 cells has been demonstrated and shown to be mediated through inhibition of ROS generation and Ca^2+^ influx [293]. Ginkgolide K (**50**) has also shown to reduce the volume of infarction and brain water content and improve neurological deficit score in ischemia-reperfusion-induced cerebral injury. It also reversed the level of MDA, NO, NOS, and SOD. Neuronal injury was also significantly improved following pretreatment with ginkgolide K (**50**) [294]. Ginkgolide B (**44**) and bilobalide (**51**) have also shown to exert neuroprotection under normoglycemia, while ginkgolide B (**44**) reduces ROS species and MDA levels in both normoglycemia and hyperglycemia ischemic rats [295]. Bilobalide (**51**) has also been previously reported with multiple mechanisms of action for its neuroprotection effect, including preservation of mitochondrial ATP synthesis, inhibition of apoptotic damage induced by staurosporine or by serum-free medium, suppression of hypoxia-induced membrane deterioration in the brain, and actions of increasing the expression of the mitochondrial DNA-encoded COX III subunit of cytochrome* c* oxidase and the NADH dehydrogenase subunit 1 [296]. Ginkgolide B (**44**) also inhibits the 6-OHDA-induced apoptosis of PC12 by upregulating the calbindin D28K mRNA and by decreasing the intracellular calcium concentration [297] and protects against ischemic stroke [298]. Ginkgolide B (**44**) has also been reported to attenuate the A*β*_(1–42)_-induced oxidative damage and altered cellular responses in human neuroblastoma (SH-SY5Y) cells [299].

#### 6.3.3. Sesquiterpenes

**Figure 9:**
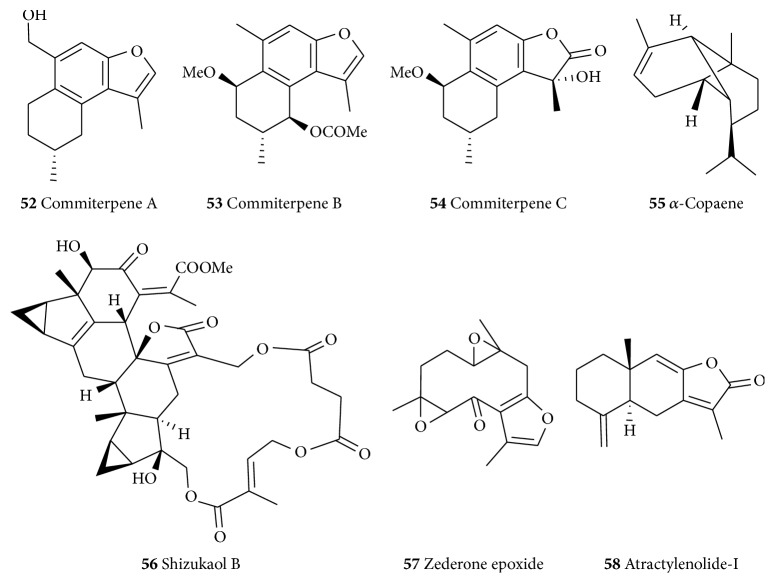
Structures of selected neuroprotective sesquiterpenes.

Three cadinane sesquiterpenes, commiterpenes A–C (**52**–**54**, Figure 9) isolated from the resinous exudates of* Commiphora myrrha* (Burseraceae), were reported to show neuroprotective effects against the MPP^+^-induced neuronal cell death in SH-SY5Y cells [300]. Also, tricyclic sesquiterpene *α*-copaene (**55**) prevents the H_2_O_2_-induced neurotoxicity [301]. Shizukaol B (**56**) is a lindenane-type dimeric sesquiterpene isolated from* Chloranthus henryi* (Chloranthaceae). It has anti-inflammatory effects in the LPS-activated microglia partly by modulating JNK-AP-1 signaling pathway. Shizukaol B (**56**) has been shown to suppress the expression of iNOS and COX-2 and production of NO, TNF-*α*, and IL-1*β* in LPS-stimulated BV2 microglia. It also inhibits the LPS-mediated JNK 1/2 activation and significantly blocks the LPS-induced AP-1 activation [302]. Many other structurally related dimeric sesquiterpenes (e.g., chloramultilide A and spicachlorantin B) along with others including zederone epoxide (**57**) have been isolated from* C. henryi* with antineuroinflammatory effects. They showed significant antineuroinflammatory effects by inhibiting NO production in the LPS-stimulated murine BV-2 microglial cells with relatively low cytotoxicity [303]. Atractylenolide-I (**58**) is isolated from the rhizomes of* Atractylodes macrocephala* (Asteraceae) a common medicinal plant in Chinese traditional medicine. The compound has been reported to reverse the MPTP-induced behavioral deficits, decreased microglial activation, and conferred protection to dopaminergic neurons in the mouse model of PD [304]. A number of other sesquiterpenes' neuroprotection [305] prevents A*β*_(25–35)_-induced toxicity in mouse cortical neurons and scopolamine-induced cognitive impairment in mice [306].

#### 6.3.4. Monoterpenes

**Figure 10:**
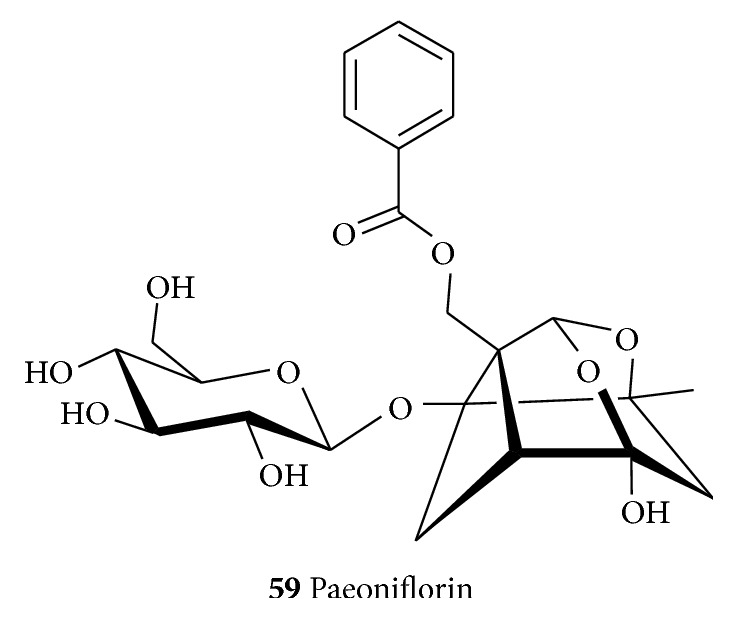
The structure of the monoterpenoid, paeoniflorin.

Paeoniflorin (**59**, Figure 10) obtained from* Paeoniae alba Radix *(Paeoniaceae) [307] has been shown to protect striatal nerve fibers and TH-positive neurons in SN; mitigate bradykinesia observed in MPTP model of PD; and ameliorate dopaminergic neurodegeneration [308]. The neuroprotective and antineuroinflammatory effects of paeoniflorin (**59**) can also be linked with the activation of adenosine A1 receptor [308]. In the 6-OHDA-induced unilateral striatal lesion in rats, it reduced apomorphine-induced rotation [309]. It also protected PC12 cells from MPP+ and acid-induced damage; reduced the influx of Ca^2+^ and its cytosolic content; upregulated microtubule-associated protein 1A/1B-light chain 3-phosphatidylethanolamine conjugate protein, and inhibited the MPP+-induced overexpression of lysosome-associated membrane protein 2a [310].

## 7. General Summary and Conclusions

A number of people living in developing countries rely on herbal medicines not only because they are considered safe but the costs associated with modern medicines are beyond the reach of many people. The growing prevalence of complex metabolic and neurodegenerative diseases in the western societies that have no drugs of cure also means that plant medicines could still be exploited as a valuable source of lead compounds as has been done throughout the history of mankind. The various biologically active secondary metabolites belonging to the polyphenols, primarily the shikimic acid and/or acetate pathways origin along with other biosynthetic pathways, and other classes of compounds including the alkaloids and terpenoids have been documented from numerous genera of plants. In this communication, these classes of compounds have been systematically presented to give an overview of plant-derived neuroprotective agents acting through diverse mechanisms of action(s). We have provided an account of some neurodegenerative diseases, some important factors in their pathogenesis, and exemplary neuroprotective drugs isolated from medicinal plants that have been used in different traditional systems of medicine.

**Figure 11:**
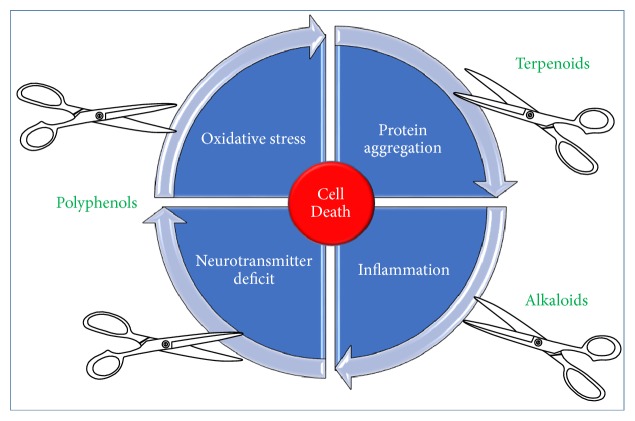
*Major targets for plant-derived neuroprotective agents*. Cutting the cycle of neuronal cell death through general anti-inflammatory and antioxidant mechanisms has been shown to play the key role in neuroprotection by natural products. Ameliorating the deleterious effect of protein aggregation such as A*β* and reversing the transmitter deficit associated with NDs through multiple mechanisms have been well documented for many plant-derived neuroprotective agents.

The interlinking mechanisms of oxidative stress and inflammation in neurodegenerative diseases have been shown to play key role in processes leading to neuronal cell death. Perhaps the most important feature of the selected natural products in this communication is their multifunctional nature that seems to give the compounds significant potency through antioxidant and anti-inflammatory mechanisms among others. Associated with these mechanisms are the other linking pathological hallmarks of NDs where protein precipitation/aggregation play pivotal role in inducing the oxidative stress and unregulated inflammation that are associated with neuronal deletions in specific areas of the brain. The overall common mechanisms of action for plant-derived neuroprotective agents are depicted in Figure 11. On the basis of the plethora of evidences presented for the plant-derived neuroprotective agents, further research on details of efficacy studies especially on human subjects is well warranted.

## Abbreviations

6-OHDA: 6-Hydroxydopamine
A*β*: Amyloid *β*
ACh: Acetylcholine
AChE: Acetylcholinesterase
AD: Alzheimer's disease
AKt: Protein kinase B
ALS: Amyotrophic lateral sclerosis
AMPA: *α*-Amino-3-hydroxy-5-methyl-4-isoxazolepropionic acid
AMPK: AMP activated protein kinase
AP-1: Activator protein-1
APP: Amyloid precursor protein
ASC: Apoptosis-associated speck-like protein containing a CARD
BACE1: Beta-secretase 1
Bax: BCL-2 associated X
Bcl-2: B-cell lymphoma 2
BuChE: Butyrylcholinesterase
CAT: Catalase
ChAT: Choline acetyl transferase
CNS: Central nervous system
COX2: Cyclooxygenase 2
DA: Dopamine
DAMPs: Ganger associated molecular pattern
DMT1-IRE: Divalent metal transporter 1 with iron responsive element
DMTI: Divalent metal transporter 1
ERK 1/2: Extracellular signal-regulated kinases 1/2
GABA: *γ*-Aminobutyric acid
GSH: Glutathione, reduced form
GSK-3*β*: Glycogen synthase kinase 3 beta
H_2_O_2_: Hydrogen peroxide
HD: Huntington's disease
HIF-1*α*: Hypoxia-inducible factor 1*α*
HO-1: Heme oxygenase 1
IGF1: Insulin-like growth factor-1
IKK-*β*: Inhibitor of nuclear factor kappa-B kinase subunit beta
IL: Interleukin
INOS: Inducible nitric oxide synthase
IPA: Indole-3-propionic acid
IRE: Iron responsive elements
JNK: C-Jun-terminal kinase
LDH: Lactate dehydrogenase
LDL: Low density lipoprotein
LPS: Lipopolysaccharide
MAO: Monoamine oxidase
MAPK: Mitogen-activated protein kinase
MDA: Malondialdehyde
MMP: Matrix metalloproteinases
MPP+: l-Methyl-4-phenylpyridinium
MPTP: 1-Methyl-4-phenyl-l,2,3,6-tetrahydropyridine
nAChR: Nicotinic acetylcholine receptors
ND: Neurodegenerative disease
NF*κ*-B: Nuclear factor kappa-light-chain-enhancer of activated B cells
NLRP3: NLR family pyrin domain containing 3
NMDA: * N*-Methyl-D-aspartate
NO: Nitric oxide
Nrf2: Nuclear factor erythroid 2-related factor 2
NTFs: Neurofibrillary tangles
^•^OH: Hydroxyl radical
O_2_^•−^: Superoxide radicals
PAMPs: Pathogenic associated molecular pattern
PARP: Poly-ADP-ribose polymerase
PD: Parkinson's disease
PRRs: Pattern recognition receptors
PUFA: Polyunsaturated fatty acid
ROS: Reactive oxygen species
SIRT: Sirtuin
SN: Substantia nigra
SOD: Superoxide dismutase
TGF: Transforming growth factor
TH: Tyrosine hydroxylase
TLRs: Toll-like receptors
TNF*α*: Tumour necrosis factor alpha.
